# Treatment setting and buprenorphine discontinuation: an analysis of multi-state insurance claims

**DOI:** 10.1186/s13722-024-00450-0

**Published:** 2024-03-16

**Authors:** Kevin Y. Xu, Alex K. Gertner, Shelly F. Greenfield, Arthur Robin Williams, Richard A. Grucza

**Affiliations:** 1 Department of Psychiatry, Health and Behavior Research Center, Washington University School of Medicine, Renard Hospital 3007A, 4940 Children’s Place, Saint Louis, MO 63110 USA; 2 University of North Carolina Hospitals, Chapel Hill, NC USA; 3 Division of Women’s Mental Health and Division of Alcohol, Drugs, and Addiction, McLean Hospital, Belmont, MA USA; 4 Department of Psychiatry, Harvard Medical School, Boston, MA USA; 5 Department of Psychiatry, College of Physicians and Surgeons, Columbia University, New York, NY USA; 6 Division of Substance Use Disorders, New York State Psychiatric Institute, New York, NY USA; 7 Advanced Health Data Institute, Department of Health and Outcomes Research, Department of Family/Community Medicine, Saint Louis University School of Medicine, St. Louis, MO USA

## Abstract

**Background:**

Potential differences in buprenorphine treatment outcomes across various treatment settings are poorly characterized in multi-state administrative data. We thus evaluated the association of opioid use disorder (OUD) treatment setting and insurance type with risk of buprenorphine discontinuation among commercial insurance and Medicaid enrollees initiated on buprenorphine.

**Methods:**

In this observational, retrospective cohort study using the Merative MarketScan databases (2006–2016), we analyzed buprenorphine retention in 58,200 US adults with OUD. Predictor variables included insurance status (Medicaid vs commercial) and treatment setting, operationalized as substance use disorder (SUD) specialty treatment facility versus outpatient primary care physicians (PCPs) versus outpatient psychiatry, ascertained by linking physician visit codes to buprenorphine prescriptions. Treatment setting was inferred based on timing of prescriber visit claims preceding prescription fills. We estimated time to buprenorphine discontinuation using multivariable cox regression.

**Results:**

Among enrollees with OUD receiving buprenorphine, 26,168 (45.0%) had prescriptions from SUD facilities without outpatient buprenorphine treatment, with the remaining treated by outpatient PCPs (n = 23,899, 41.1%) and psychiatrists (n = 8133, 13.9%). Overall, 50.6% and 73.3% discontinued treatment at 180 and 365 days respectively. Buprenorphine discontinuation was higher among enrollees receiving prescriptions from SUD facilities (aHR = 1.03[1.01–1.06]) and PCPs (aHR = 1.07[1.05–1.10]). Medicaid enrollees had lower buprenorphine retention than those with commercial insurance, particularly those receiving buprenorphine from SUD facilities and PCPs (aHR = 1.24[1.20–1.29] and aHR = 1.39[1.34–1.45] respectively, relative to comparator group of commercial insurance enrollees receiving buprenorphine from outpatient psychiatry).

**Conclusion:**

Buprenorphine discontinuation is high across outpatient PCP, psychiatry, and SUD treatment facility settings, with potentially lower treatment retention among Medicaid enrollees receiving care from SUD facilities and PCPs.

**Supplementary Information:**

The online version contains supplementary material available at 10.1186/s13722-024-00450-0.

## Introduction

Buprenorphine is underutilized in the U.S. despite its effectiveness in reducing opioid use disorder (OUD)-related overdose and mortality [1–3]. Differences in buprenorphine utilization and treatment outcomes across different outpatient provider and setting types are poorly characterized in multi-state data. Amid a commonly held perception that patients with OUD are psychiatrically complex and difficult to treat, concerns have also been described about potentially inferior outcomes when buprenorphine is prescribed by clinicians without mental health expertise or access to psychiatric consultants [4]. Physicians have commonly cited a perceived lack of knowledge and experience in prescribing buprenorphine [4], with health care professionals expressing concern about diversion risk and difficulty titrating dose [5]. A recent study of North Carolina Medicaid claims by Gertner and colleagues showed that primary care physicians and specialists may deliver comparable buprenorphine treatment quality among Medicaid beneficiaries [6]. In light of a relative dearth of buprenorphine prescribers who accept Medicaid and potential barriers to buprenorphine retention in commercial insurance enrollees [7, 8], no multi-state studies in the US, differentiating between Medicaid and commercial insurance have compared treatment characteristics in SUD specialty facilities versus outpatient psychiatry and primary care settings. The ability to differentiate between Medicaid and commercial insurance is important, as insurance status is likely to influence buprenorphine access; this is evidenced by 2017 data showing greater prior authorization barriers for buprenorphine access among Medicaid enrollees compared to commercially-insured peers [9]. A recent thematic analysis showed that most U.S. Medicaid plans required prior authorization for at least 1 buprenorphine formulation, in addition to having a high burden of restrictive surveillance, behavioral health treatment mandates, and dosage caps [10].

To address this gap, we used data from a multi-state cohort of commercial insurance and Medicaid enrollees receiving OUD treatment to evaluate the association of treatment setting (substance use disorder [SUD] facility versus outpatient primary care versus outpatient psychiatry) with treatment discontinuation rates among enrollees initiated on buprenorphine. Amid concern for disparities in buprenorphine access by insurance status, we analyzed the relationship between potential setting type by insurance interactions as a secondary aim.

## Methods

### Study overview

This retrospective cohort study categorized buprenorphine prescriptions by setting type. This was accomplished by linking patients’ outpatient primary care, outpatient psychiatry, and SUD facility prescriber visits with patients’ pharmacy claims buprenorphine prescriptions by temporal proximity (meaning, new buprenorphine fills 14 days following a physicians’ visit containing data on setting type). Data were obtained from the Merative MarketScan Commercial Claims and Encounters and Medicaid databases. As previously described [11], the MarketScan databases contains claims for individuals in the U.S., ages 16–64 years for prescription data (quantity, dosing, and fill dates), diagnoses, and longitudinal information on inpatient and outpatient health care utilization. Our data were available from January 1, 2006 to December 31, 2016. Strengthening the Reporting of Observational Studies (STROBE) and RECORD-PE reporting guidelines were followed for this study. This study was exempted from review by the Washington University Institutional Review Board.

### Participants and variables

**Fig. 1:**
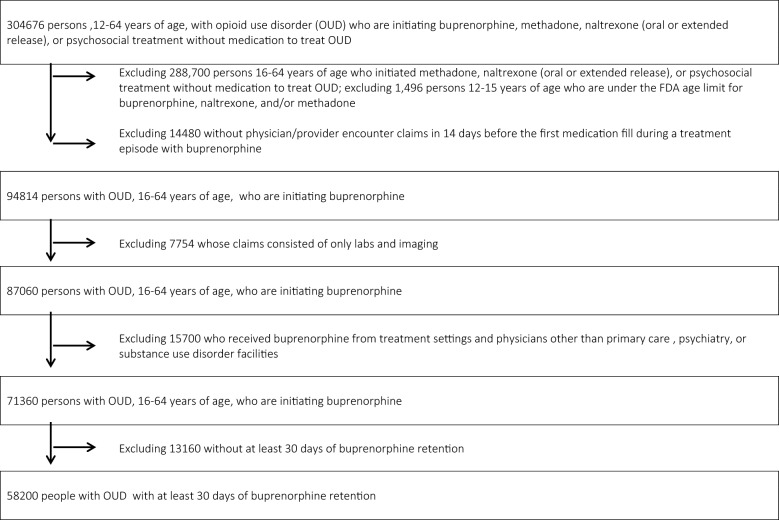
Derivation of the analytic sample

As shown in Fig. 1, our analytic sample was derived from a cohort of 304,676 individuals in the US with > 6 months of continuous insurance enrollment preceding *initiation of buprenorphine* (index event, time = 0) for collection of covariates, prescription drug coverage, and an OUD diagnosis. We required two instances of an OUD diagnostic code on a claim in order to increase the specificity of OUD ascertainment [12]: (1) an ICD-9/10 diagnostic code on a claim for opioid “dependence or abuse” and (2) with an additional second claim pertaining to the receipt of treatment for opioid “dependence or abuse” (medication for OUD [MOUD] [buprenorphine, methadone, naltrexone], psychosocial services such as case management, behavioral health screening, or psychological counseling. (The “dependence or abuse” term is still employed in the ICD codes despite the stigmatizing nature of the term “abuse”). We excluded 1,496 persons 12–15 years of age (under the FDA age limit for MOUD prescribing), 288,700 persons 16–64 years of age who did not receive MOUD, 14,480 without data on physician/prescriber encounters for OUD treatment in the 14 days before the first medication fill during a treatment episode with buprenorphine. We also excluded 7,754 enrollees whose visits were for solely labs and imaging and 15,700 who received buprenorphine from inpatient hospital settings. Finally, we excluded 13,160 without at least 30 days of buprenorphine retention, because we were interested in longer-term buprenorphine retention in the outpatient setting, beyond the initial induction period. This culminated in 58,200 enrollees with OUD with at least 30 days of buprenorphine retention in our final analytic sample.

The primary outcome was time until buprenorphine discontinuation, operationalized as days until discontinuation of treatment (defined as a gap of at least 45 days in buprenorphine medication possession). Previous studies have found that different lengths of the gap used to define buprenorphine discontinuation may impact results [13], as some studies have used 30-day [14] whereas others have used 60-day thresholds [15].. Recognizing a lack of consensus in the gap duration threshold for buprenorphine discontinuation, we elected to use a cutoff of 45 days, as done in other analyses of this cohort [16].

The primary predictor variable was setting type. Because insurance claims contain data on medications filled by patients, rather than orders (prescriptions written by a provider that may or may not be filled by the patient), we inferred setting-type based on new buprenorphine prescriptions filled within 14 days following an OUD treatment encounter with a prescribing practitioner. We followed the methods used by prior claims-based studies, which have inferred indication by examining provider visit codes occurring prior to prescription fills [17]. Recognizing that patients may experience delays in filling buprenorphine beyond 14 days [18], sensitivity analyses that employed a buprenorphine fill-health care professional visit linkage window of 30 days as opposed to 14 days were conducted. As shown in the eMethods, our observation window for buprenorphine prescription encounters was thus the 14 days preceding an enrollee’s first buprenorphine prescription fill, meaning that we observed all physician visit claims with an OUD diagnosis during the 14 days prior to the prescription fill and coded their SUD facility setting to link prescriber visits to buprenorphine scripts. As shown in Additional file 1: Table S1, treatment setting was operationalized as the following categories: (1) SUD treatment facility; (2) outpatient primary care; and (3) outpatient psychiatry, with the classification scheme depicted in the Additional file 1. In brief, we used the Centers for Medicare and Medicaid Services place-of-service code (STDPLAC) and the proprietary MedSTAT service category variable (SVCSCAT) to limit each claim in the 14-day look-back period to either SUD facilities or outpatient encounters with a prescriber, which we limited to outpatient psychiatry or outpatient primary care medicine (i.e. internal medicine, family medicine, geriatrics, pediatrics). We thus classified each claim in the 14-day look-back period into 3 categories: (1) SUD facility; (2) outpatient psychiatry; and (3) outpatient primary care medicine. For enrollees who had multiple linkages, we selected the most common of our 3 settings (based on number of encounters) during the 14 days surrounding treatment initiation. For persons who had a tie among the most common treatment setting, we classified enrollees to their treatment setting category based on their last encounter (closest to the time of treatment initiation).

As shown in Additional file 1: Table S2, Covariates included insurance status (Medicaid vs. commercial), race/ethnicity (data available only for Medicaid enrollees), co-occurring substance use disorders (alcohol, cocaine, amphetamine, sedative) and mood, anxiety, and psychotic disorders in the 6 months prior to the initiation of treatment, with these conditions measured as dichotomous variables and identified from inpatient and outpatient claims using ICD-9/10 codes. We also included emergency admissions or inpatient hospitalizations for drug-related poisoning using CDC and Safe State Alliance’s consensus recommendations for poisoning and pain, which encompass acute care encounters involving opioids as well as other types of substances commonly implicated in opioid-related adverse events such as poisoning secondary to opioids together with benzodiazepines and/or alcohol [19, 20].

### Statistical analysis

We first calculated descriptive statistics to compare demographic and clinical characteristics between different treatment settings, using chi-square and Kruskal–Wallis tests. We then conducted univariate analyses computing the percentage of enrollees who were retained in treatment at 180- and 365-days. Next, we used multivariable survival analysis (cox proportional hazards) methods techniques to assess the association of treatment setting with time (days) until buprenorphine discontinuation or censoring (end of eligibility), beginning at 30-days post-treatment initiation (beginning of observation window for the cox regression). Our cox models adjusted for covariates (past 6-month drug-related poisonings, co-occurring psychiatric disorders, co-occurring substance use disorders). We evaluated the proportionality of hazards assumption using Schoenfeld residuals and Loess plots, which were satisfied. We calculated variance inflation factors to assess multicollinearity, finding no significant collinearity using a threshold of < 2.0.

We sought to test the robustness of the Cox regression models. We conducted sensitivity analyses specifying discontinuation with 30- and 60-day gaps in lieu of 45-day gaps. As an additional sensitivity analysis, we used 30 days preceding buprenorphine initiation to link physician visits and buprenorphine fills. We conducted analyses controlling for buprenorphine dose in our models (specifically the percentage of enrollees receiving doses greater than 16 mg and 24 mg daily), as higher dosages may mediate improved treatment retention [21], with recent data showing that enrollees prescribed the recommended daily dose of buprenorphine (16 mg) were less likely to be retained in treatment at 180-days than those prescribed a higher dose (specifically 24 mg daily) [22].

As a secondary analysis, we evaluated potential interactions between Medicaid status and setting type with regards to buprenorphine retention, creating a six-level variable reflecting all combinations of setting type and insurance status (SUD facility + Medicaid; SUD facility + commercial; PCP + Medicaid; PCP + commercial; psychiatry + Medicaid; psychiatry + commercial insurance). Analyses were conducted from June 2, 2021 through June 28, 2023. Two-sided P-values were applied using SAS Version 9.4.

## Results

### Demographic and clinical characteristics

Participant demographic and clinical characteristics are depicted in Table 1. Our sample consisted of 58,200 enrollees with OUD (mean age, 34.8 [SD 11.6] years; 25,947 [44.6%] female). Among Medicaid enrollees, 625 [5.3%] were non-Hispanic Black, 157 [1.3%] Hispanic, and 9,560 [81.2%] non-Hispanic White, with the remainder classified as “other/missing”; race/ethnicity is not provided by commercial insurance claims in the Merative MarketScan data.

**Table 1:** Clinical and demographic characteristics

	ALL, n = 58,200	SUD Facility, n = 26,168	Outpatient Primary Care, n = 23,899	Outpatient Psychiatry, n = 8,133	P
	n (%)	n (%)	n (%)	n (%)	
Demographic variables					
Sex					** < 0.001**
Male sex	32,253 (55.4)	14,736 (56.3)	12,600 (52.7)	4917 (60.5)	
Female sex	25,947 (44.6)	11,432 (43.7)	11,299 (47.3)	3216 (39.5)	
Mean age (SD	34.8 (11.6)	33.0 (10.6)	37.2 (12.0)	33.4 (11.6)	** < 0.001**
Age over 30 years	34,737 (59.7)	14,205 (54.3)	16,238 (67.9)	4294 (52.8)	** < 0.001**
Insurance					** < 0.001**
Medicaid	12,287 (21.1)	6640 (25.4)	4841 (20.3)	806 (9.9)	
Commercial	45,913 (78.9)	19,528 (74.6)	19,058 (79.7)	7327 (90.1)	
Race/Ethnicity (among medicaid only, n = 12,287)					**0.01**
NH White	9560 (81.2)	5216 (81.5)	3670 (80.0)	674 (85.6)	
NH Black	625 (5.3)	331 (5.2)	258 (5.6)	36 (4.6)	
Hispanic	157 (1.3)	82 (1.3)	71 (1.6)	4 (0.5)	
Other	1435 (12.2)	775 (12.1)	587 (12.8)	73 (9.3)	
Co-occurring substance use characteristics in 6 months prior to buprenorphine initiation					
> 1 admission for drug-related poisoning in the 6 months prior to buprenorphine initiation	5130 (8.8)	2151 (8.2)	2219 (9.3)	760 (9.3)	** < 0.001**
Alcohol use disorder	4943 (8.5)	2163 (8.3)	1930 (8.1)	850 (10.5)	** < 0.001**
Sedative use disorder	3118 (5.4)	1369 (5.2)	1276 (5.3)	473 (5.8)	**0.12**
Cocaine use disorder	2400 (4.1)	1160 (4.4)	896 (3.8)	344 (4.2)	**0.001**
Methamphetamine use disorder	1396 (2.4)	738 (2.8)	471 (2.0)	187 (2.3)	** < 0.001**
Co-occurring non-SUD psychiatric disorder in 6 months prior to buprenorphine initiation					
Anxiety disorder	17,953 (30.9)	8116 (31.1)	7332 (30.7)	2505 (30.8)	0.72
Mood disorder	21,004 (36.1)	8832 (33.8)	3523 (35.7)	3659 (44.9)	** < 0.001**
Psychotic disorder	915 (1.6)	361 (1.4)	394 (1.7)	160 (2.0)	**0.001**
Charlson comorbidity index (CCI) based on comorbidities in the 6 months prior to buprenorphine initiation					
CCI = 0	53,028 (91.1)	24,468 (93.5)	20,972 (87.8)	7588 (93.3)	
CCI = 1 or 2	4704 (8.1)	1558 (6.0)	2641 (11.1)	504 (6.2)	
CCI = 3 +	468 (0.8)	141 (0.5)	286 (1.2)	41 (0.5)	
Buprenorphine treatment characteristics					
Mean daily dose of 16 + mg of buprenorphine	12,930 (22.2)	5681 (21.7)	5626 (23.5)	1623 (20.0)	** < 0.001**
Mean daily dose of 24 + mg of buprenorphine	3589 (6.2)	1698 (6.5)	1505 (6.3)	386 (4.8)	** < 0.001**
Buprenorphine discontinuation at 180 days	29,423 (50.6)	13,129 (50.2)	12,208 (51.1)	4086 (50.2)	0.11
Buprenorphine discontinuation at 365 days	42,653 (73.3)	19,323 (73.8)	17,545 (73.4)	5785 (71.1)	** < 0.001**

P-values were bolded to reflect statistical significance, p < .05

Overall, 26,168 (45%) had buprenorphine prescriptions from SUD facilities. Among those who received buprenorphine from outpatient settings, 23,899 were treated by primary care physicians (PCPs) and 8133 by psychiatrists. We observed a significant association between insurance status and treatment setting (p < 0.001). 21.1% (n = 12,287) was enrolled in Medicaid overall. Yet, of the 26,168 enrollees who received buprenorphine from SUD facilities, 25.4% (n = 12,287) were enrolled in Medicaid; of the 23,899 enrollees who received buprenorphine from outpatient primary care providers, 4829 (20.3%) were enrolled in Medicaid. In comparison, 806 of the 8,133 (9.9%) who received buprenorphine from outpatient psychiatry were enrolled in Medicaid.

Among Medicaid enrollees (because race/ethnicity data is only available for Medicaid enrollees), 85.6% (n = 674 out of 8133) of those who received buprenorphine from psychiatrists were non-Hispanic White, compared to 81.5% (5216 out of 26,168) and 80% (3670 out of 23,899) in the SUD facility and outpatient PCP cohorts (p = 0.01) respectively. Only a minority of enrollees were diagnosed with a co-occurring substance use disorder in the 6 months prior to buprenorphine initiation, with the prevalence of comorbid alcohol, stimulant, and sedative use disorders each under 10%; however, 17,953 (30.9%) had an anxiety disorder diagnosis and 21,004 (36.1%) had a mood disorder diagnosis. Approximately 9% (n = 5130) of the sample had an admission for drug-related poisoning in the 6 months preceding buprenorphine initiation.

### Treatment characteristics by setting type

As depicted in Table 1, with regard to treatment retention, 29,423 (51%) discontinued buprenorphine at 180 days, with similar percentages among outpatient treatment settings and SUD facilities (13,129 [50.2%] for SUD facilities; 12,208 [51.1%] for outpatient primary care; 4086 [50.2%] for outpatient psychiatry, p = 0.11). At 365 days, 42,653 (73%) overall discontinued buprenorphine, with slightly lower discontinuation rate seen among enrollees receiving buprenorphine from outpatient psychiatry (n = 5785, 71.1%) versus 73.8% (n = 19,323) for SUD facilities and 73.4% (n = 17,545) for outpatient primary care (p < 0.001).

As shown in Table 2, in adjusted cox proportional hazards models, we observed a modestly higher treatment discontinuation among enrollees receiving buprenorphine from SUD facilities (aHR = 1.03 [1.01–1.06]) and outpatient primary care physicians (aHR = 1.08 [1.05–1.10]) after controlling for commercial insurance status (a protective factor against treatment discontinuation, aHR = 0.80[0.78–0.82], Additional file 1: Table S3), demographics and other clinical characteristics. Similar findings were observed in analyses of the Medicaid subgroup, controlling for race/ethnicity (race/ethnicity data is not available in the commercial enrollee cohort). As a sensitivity analysis, we computed models that employed 30- and 60-day gaps between buprenorphine episodes, illustrating similar effects as parent analyses (Additional file 1: Table S3). Likewise, our results were robust in sensitivity analyses that employed a buprenorphine fill-health care professional visit linkage window of 30 days as opposed to 14 days (Additional file 1: Table S4). Because buprenorphine dose has been found to be a mediator of retention (i.e. higher doses with longer retention), we examined whether variation in dosage by health care professional type could explain these results. Across all enrollees, 12,930 (22.2%) received at least 16 mg of buprenorphine daily during treatment, with 3,589 (6.2%) receiving at least 24 mg daily. Outpatient psychiatry setting had lower rates of 24 + mg buprenorphine receipt than other settings (n = 1698 [6.5%] for SUD facility; n = 1505 [6.3%] for outpatient primary care; n = 383 [4.8%] for outpatient psychiatry, p < 0.001). Yet, inclusion of buprenorphine dose in the proportional hazards models did not significantly impact the results (Additional file 1: Table S5).

**Table 2:** Hazard Ratios depicting time to buprenorphine discontinuation or censoring

		aHR	95% CI	
Model 1	Substance Use Disorder Facility vs. Outpatient Psychiatry	1.03	1.01	1.06
	Outpatient Primary Care vs Outpatient Psychiatry	1.08	1.05	1.10
Model 2 Limited to Medicaid enrollees (adjusting for race/ethnicity)	Substance Use Disorder Facility vs. Outpatient Psychiatry	1.21	1.12	1.30
	Outpatient Primary Care vs Outpatient Psychiatry	1.36	1.26	1.47
Model 3	SUD Facility + Medicaid (vs Outpatient Psychiatry + Commercial Insurance)	1.24	1.20	1.29
	SUD Facility + Commercial Insurance (vs Outpatient Psychiatry + Commercial Insurance)	1.02	0.99	1.05
	Outpatient Primary Care + Medicaid (vs Outpatient Psychiatry + Commercial Insurance)	1.39	1.34	1.45
	Outpatient Primary Care + Commercial Insurance (vs Outpatient Psychiatry + Commercial Insurance)	1.04	1.01	1.07
	Outpatient Psychiatry + Medicaid (vs Outpatient Psychiatry + Commercial Insurance)	1.03	0.96	1.11

This table illustrates multivariable cox regression models, with aHR representing adjusted hazard ratios. An aHR greater than 1 represents an association of setting type with higher likelihood of discontinuation; an aHR less than 1 represents an association of setting type with lower likelihood of discontinuation; an aHR of precisely 1 would represent no association between setting type and discontinuation

Full Models are shown in the Supplementary Information. Model 1 adjusts for: 1) Male vs Female, 2) Commercial vs Medicaid, 3) Age > 30 vs < 30 years, 4) Co-occurring alcohol use disorder vs no alcohol use disorder, 5) Co-occurring amphetamine use disorder vs no amphetamine use disorder, 6) Co-occurring cocaine use disorder vs no cocaine use disorder, 7) Co-occurring sedative use disorder vs no sedative use disorder, 8) Co-occurring mood disorder vs no mood disorder, 9) Co-occurring anxiety disorder vs no anxiety disorder, 10) Co-occurring psychotic disorder vs no psychotic disorder, 11) Charlson comorbidity index = 1 or 2 vs charlson comorbidity index = 0, 12), charlson comorbidity index = 3 + vs charlson comorbidity index = 0, 13) Drug-related poisoning in the 6 months preceding treatment initiation. Model 2 adjusts for: 1) Male vs Female, 2) Race/Ethnicity (non-Hispanic Black vs White; Hispanic vs White; other/unknown race vs White),, 3) Age > 30 vs < 30 years, 4) Co-occurring alcohol use disorder vs no alcohol use disorder, 5) Co-occurring amphetamine use disorder vs no amphetamine use disorder, 6) Co-occurring cocaine use disorder vs no cocaine use disorder, 7) Co-occurring sedative use disorder vs no sedative use disorder, 8) Co-occurring mood disorder vs no mood disorder, 9) Co-occurring anxiety disorder vs no anxiety disorder, 10) Co-occurring psychotic disorder vs no psychotic disorder, 11) Charlson comorbidity index = 1 or 2 vs charlson comorbidity index = 0, 12), charlson comorbidity index = 3 + vs charlson comorbidity index = 0, 13) Drug-related poisoning in the 6 months preceding treatment initiation. Model 3 adjusts for: 1) Age > 30 vs < 30 years, 2) Co-occurring alcohol use disorder vs no alcohol use disorder, 3) Co-occurring amphetamine use disorder vs no amphetamine use disorder, 4) Co-occurring cocaine use disorder vs no cocaine use disorder, 5) Co-occurring sedative use disorder vs no sedative use disorder, 6) Co-occurring mood disorder vs no mood disorder, 7) Co-occurring anxiety disorder vs no anxiety disorder, 8) Co-occurring psychotic disorder vs no psychotic disorder, 9) Charlson comorbidity index = 1 or 2 vs charlson comorbidity index = 0, 10), charlson comorbidity index = 3 + vs charlson comorbidity index = 0, 11) Drug-related poisoning in the 6 months preceding treatment initiation

### Treatment characteristics by setting and insurance type

As depicted in Additional file 1: Table S6, we computed a variable reflecting all 6 combinations of setting (SUD facility vs outpatient primary care vs outpatient psychiatry) and insurance type (Medicaid vs commercial insurance). In univariate analyses, we first observed statistically significant (p < 0.001) differences in 180-day and 365-day buprenorphine discontinuation between Medicaid and commercial insurance enrollees. Whereas 5575/12,287 (45.4%) of the Medicaid enrollees were retained in treatment at 180-days, this figure was 50.5% (n = 23,202/45,913) in the commercial insurance cohort. Furthermore, 28.6% (n = 13,141/45,913) of commercial enrollees versus 19.6% (n = 2406/12,287) of Medicaid enrollees were retained in buprenorphine at 365 days. When we examined combinations of insurance status and provider type, we found statistically significant (p < 0.001) differences in 180-day and 365-day buprenorphine discontinuation across the 6 setting-insurance combinations. Differences in 180-day retention were modest but statistically significant (p < 0.001), ranging from 42.2% (2043/4841) for outpatient primary care + Medicaid to 51.2% (413/806) for outpatient psychiatry + Medicaid (Additional file 1: Table S6). At 365 days, the differences in retention ranged from 17.2% (831/4841) for outpatient primary care + Medicaid to 29.4% (237/806) for outpatient psychiatry + Medicaid.

We subsequently conducted multivariable cox regression analyses; Table 2 shows the adjusted association of all 6 combinations of setting type and insurance type with buprenorphine retention, with the outpatient psychiatry + commercial insurance cohort serving as a reference group. Medicaid enrollees receiving buprenorphine from SUD facilities (aHR = 1.24 [1.20–1.29]) and outpatient primary care (aHR = 1.39 [1.34–1.45]) exhibited significantly higher treatment discontinuation than the outpatient psychiatry + commercial insurance reference cohort. Relative to the reference cohort, treatment discontinuation was otherwise relatively similar in all other setting type by insurance comparisons (i.e., SUD facility + commercial insurance; outpatient primary care + commercial insurance; outpatient psychiatry + Medicaid).

## Discussion

Our results show that buprenorphine discontinuation rates are high overall across multiple treatment settings, including outpatient primary care physicians, outpatient psychiatrists, and SUD facilities. Amid an urgent need for research on objective measures for long-term treatment retention such as documented buprenorphine fills [21], our study shows that half of patients were not retained in buprenorphine treatment at 180 days, with the vast majority discontinuing at one year.

Our data also illustrate that among Medicaid enrollees (a subgroup of patients who tend to have low income and are disproportionately affected by OUD), few patients with OUD see psychiatrists for buprenorphine prescriptions, which could potentially be due to a lack of access. Recognizing that Medicaid insurance, as the primary payer, can be seen as a proxy for lower socioeconomic status, our study finds that Medicaid enrollees are less likely to obtain buprenorphine via outpatient psychiatrists and more likely to receive care via SUD facilities. These findings are supported by past studies; office-based outpatient buprenorphine treatment has been found to be disproportionately accessed by enrollees using cash payment [23] or private insurance [24]. A survey data of 1,174 buprenorphine-prescribing physicians in the US found only less than half accepted Medicaid [7]. Even though the overall differences in treatment retention were modest across setting types, our analyses show that among Medicaid enrollees, substance use disorder facilities and primary care were associated with earlier treatment discontinuation than outpatient psychiatry. In contrast, the overall treatment retention metrics were similar across (1) commercial insurance enrollees receiving buprenorphine from all 3 setting types (SUD facility, primary care, psychiatry); and (2) Medicaid enrollees receiving buprenorphine from psychiatrists. The reasons for these findings are complex and warrant further investigation. The association of decreased buprenorphine retention with buprenorphine receipt from psychiatrists among Medicaid enrollees could be the consequence of access to treatment for common co-occurring disorders, such as anxiety and depression, which may otherwise interfere with OUD treatment outcomes. In other words, treatment of co-occurring mental health conditions may be associated with increased treatment engagement and retention in patients initiating buprenorphine, especially in Medicaid patients. However, in Medicaid enrollees with OUD, access to comprehensive mental health treatment appears to be the exception, rather than the norm. In a national survey of addiction treatment programs serving a largely publically-insured cohort [25], only 1% routinely accepting patients with co-occurring disorders regardless of severity [26]. Furthermore, Medicaid enrollees who obtain buprenorphine from psychiatrists have been found to have fewer physical health needs interfering with treatment and better social and financial support, contributing to better buprenorphine retention [27]. Finally, some psychiatrists may select for patients who are more likely to be retained through office policies or terminate patients who struggle with treatment adherence [28]. This study has several limitations. First, our study is limited to enrollees who filled buprenorphine prescriptions after seeing outpatient or SUD facility providers and who were retained in OUD treatment for at least 30 days. We cannot assume that the treatment setting during the buprenorphine initiation will be consistent for the entire duration of treatment, which may bias the results towards the null. Furthermore, these results do not generalize to individuals who received buprenorphine from inpatient-based settings and do not shed light on differences in buprenorphine initiation outcomes in the hospital setting (i.e., differences across addiction psychiatry and medicine consult services). Particularly since we restricted to only enrollees who had 30 days of retention and selected enrollees who received outpatient treatment, these analyses may be considered a “best case scenario” cohort; especially since people who are uninsured or incarcerated are not present in the MarketScan dataset, administrative databases like MarketScan are likely to underrepresent racially minoritized groups, limiting the study’s generalizability.

Second, measurement error cannot be ruled out as medication prescription fills reflected in insurance claims data may not reflect actual patient use; for instance, a patient may successfully fill their buprenorphine but may frequently miss doses that are not captured in the MarketScan data. Third, confounding cannot be ruled out such that the populations receiving care from psychiatrists and primary care physicians may differ such that those in treatment with a psychiatrist may have self-selected for instance, because of depression or anxiety symptoms, for example. However, we sought to mitigate such confounding via adjustment in our models, such as by controlling for admissions for drug-related poisonings in the 6-months preceding treatment initiation as a marker for stability. While our study is strengthened by its multi-state Medicaid sample, our Medicaid subset does not cover every state in the US, and our data does not include Medicare or self-pay. Future studies should investigate regional disparities [29] in our outcomes, as seeing a psychiatrist may be more concentrated among those with means as demonstrated by enrollment in commercial insurance and also in regions where psychiatrists are present. Further geographic heterogeneity in buprenorphine access is suggested by recent thematic analysis [10] of state Medicaid prior authorization requirements, showing substantial heterogeneity in buprenorphine dosages requiring prior authorizations.

The MarketScan databases also lack detailed information on racial/ethnic demographics; given the crisis of structural racism contributing to disparities in OUD treatment, focused research should be conducted on high treatment discontinuation rates in minoritized populations. Amid rising overdose deaths in reproductive-age women, who have a significantly higher rate of co-occurring mood and anxiety disorders [30, 31], research on the intersection of sex, gender, and SUD treatment are desperately needed [32–36], and this analysis should also be replicated in perinatal and postpartum populations receiving care in obstetric care settings. Finally, the age of the dataset (2006–2016), predating the recent surge in fentanyl and potency-enhancing synthetics, poses a limitation to studying buprenorphine treatment outcomes associated; our study was unable to evaluate treatment outcomes associated with buprenorphine prescriptions written by nurse practitioners and physician assistants because the Comprehensive Addiction Recovery Act, was only signed into law in July 2016 and enacted in 2017 [37].

## Conclusions

In conclusion, despite the efficacy of buprenorphine treatment in OUD [1], our study shows there is significant room for improvement with regard to buprenorphine discontinuation rates in the US with low rates of long-term retention irrespective of health care professional type. Although retention rates were mildly better in the outpatient psychiatry cohort, this cohort serves a lower proportion of Medicaid enrollees, and rates remained poor across SUD facilities, outpatient psychiatrists, and outpatient primary care physicians. Even though Medicaid enrollees receiving buprenorphine from psychiatry performed as well as commercial enrollees with regards to retention only a small fraction of Medicaid enrollees received buprenorphine from outpatient psychiatry.

### Supplementary Information

**Additional file 1: Methods S1.** Design Diagram. **Table S1.** Classification of Setting Type. **Table S2.** Administrative codes for classification of covariates. **Table S3.** Treatment Setting Type and Time to Buprenorphine Discontinuation, Sensitivity Analyses by Length of Buprenorphine Episode Gap. **Table S4.** Treatment Setting Type and Time to Buprenorphine Discontinuation, Sensitivity Analyses by Length of Prescription-Prescriber Visit Linkage. **Table S5.** Treatment Setting Type and Time to Buprenorphine Discontinuation, Controlling for Mean Buprenorphine Dose During Treatment Episode. **Table S6.** Insurance status, Setting, and 180-day and 365-day buprenorphine retention.

## Data Availability

The datasets generated during and/or analysed during the current study are not publicly available due MarketScan (proprietary) data use agreements. Data may be available from Merative MarketScan on reasonable request. We intend to provide relevant code on written reasonable request.

## References

[1] Madras BK, Ahmad NJ, Wen J, Sharfstein J (2020). Improving access to evidence-based medical treatment for opioid use disorder: strategies to address key barriers within the treatment system. National academy of medicine 2020. The prevention, treatment, and recovery working group of the action collaborative on countering the U.S. Opioid Epidemic. NAM Perspect.

[2] Xu KY, Mintz CM, Presnall N, Bierut LJ, Grucza RA (2022). Comparative effectiveness associated with buprenorphine and naltrexone in opioid use disorder and cooccurring polysubstance use. Jama Netw Open..

[3] Wakeman SE, Larochelle MR, Ameli O, Chaisson CE, McPheeters JT, Crown WH (2020). Comparative effectiveness of different treatment pathways for opioid use disorder. Jama Netw Open..

[4] Haffajee RL, Bohnert ASB, Lagisetty PA (2018). Policy pathways to address provider workforce barriers to buprenorphine treatment. Am J Prev Med.

[5] Arfken CL, Johanson CE, di Menza S, Schuster CR (2010). Expanding treatment capacity for opioid dependence with office-based treatment with buprenorphine: national surveys of physicians. J Subst Abuse Treat.

[6] Gertner AK, Robertson AG, Powell BJ, Jones H, Silberman P, Domino ME (2020). Primary care providers and specialists deliver comparable buprenorphine treatment quality. Health Aff (Millwood).

[7] Knudsen HK, Studts JL (2019). Physicians as mediators of health policy: acceptance of medicaid in the context of buprenorphine treatment. J Behav Health Ser R.

[8] Manhapra A, Agbese E, Leslie DL, Rosenheck RA (2018). Three-year retention in buprenorphine treatment for opioid use disorder among privately insured adults. Psychiat Serv.

[9] Andraka-Christou B, Simon KI, Bradford WD, Nguyen T (2023). Buprenorphine treatment for opioid use disorder: comparison of insurance restrictions, 2017–21. Health Aff.

[10] Nguemeni Tiako MJ, Dolan A, Abrams M, Oyekanmi K, Meisel Z, Aronowitz SV (2023). Thematic analysis of state medicaid buprenorphine prior authorization requirements. JAMA Netw Open.

[11] Xu KY, Borodovsky JT, Presnall N, Mintz CM, Hartz SM, Bierut LJ (2021). Association between benzodiazepine or Z-drug prescriptions and drug-related poisonings among patients receiving buprenorphine maintenance: a case-crossover analysis. Am J Psychiatry..

[12] Scherrer JF, Sullivan MD, LaRochelle MR, Grucza R (2023). Validating opioid use disorder diagnoses in administrative data: a commentary on existing evidence and future directions. Addict Sci Clin Pract.

[13] Dong HR, Stringfellow EJ, Russell WA, Bearnot B, Jalali MS (2022). Impact of alternative ways to operationalize buprenorphine treatment duration on understanding continuity of care for opioid use disorder. Int J Ment Health Ad..

[14] Meinhofer A, Williams AR, Johnson P, Schackman BR, Bao YH (2019). Prescribing decisions at buprenorphine treatment initiation: do they matter for treatment discontinuation and adverse opioid-related events?. J Subst Abuse Treat.

[15] Williams AR, Samples H, Crystal S, Olfson M (2020). Acute care, prescription opioid use, and overdose following discontinuation of long-term buprenorphine treatment for opioid use disorder. Am J Psychiat.

[16] Xu KY, Huang V, Williams AR, Martin CE, Bazazi AR, Grucza RA (2023). Co-occurring psychiatric disorders and disparities in buprenorphine utilization in opioid use disorder: an analysis of insurance claims. Drug Alcohol Depend Rep.

[17] Chua KP, Fischer MA, Linder JA (2019). Appropriateness of outpatient antibiotic prescribing among privately insured US patients: ICD-10-CM based cross sectional study. BMJ..

[18] Jakubowski A, Lu T, DiRenno F, Jadow B, Giovanniello A, Nahvi S (2020). Same-day vs. delayed buprenorphine prescribing and patient retention in an office-based buprenorphine treatment program. J Subst Abuse Treat..

[19] Lim JK, Earlywine JJ, Bagley SM, Marshall BDL, Hadland SE (2021). Polysubstance involvement in opioid overdose deaths in adolescents and young adults, 1999–2018. JAMA Pediatr.

[20] Park JN, Schneider KE, Fowler D, Sherman SG, Mojtabai R, Nestadt PS (2022). Polysubstance overdose deaths in the fentanyl era: a latent class analysis. J Addict Med.

[21] Kennedy AJ, Wessel CB, Levine R, Downer K, Raymond M, Osakue D (2022). Factors associated with long-term retention in buprenorphine-based addiction treatment programs: a systematic review. J Gen Intern Med.

[22] Chambers LC, Hallowell BD, Zullo AR, Paiva TJ, Berk J, Gaither R (2023). Buprenorphine dose and time to discontinuation among patients with opioid use disorder in the era of fentanyl. Jama Netw Open..

[23] Van Zee A, Fiellin DA (2019). Proliferation of cash-only buprenorphine treatment clinics: a threat to the nation's response to the opioid crisis. Am J Public Health.

[24] Olfson M, Zhang V, King M, Mojtabai R (2021). Changes in buprenorphine treatment after medicaid expansion. Psychiatr Serv.

[25] McGovern MP, Lambert-Harris C, Gotham HJ, Claus RE, Xie HY (2014). Dual diagnosis capability in mental health and addiction treatment services: an assessment of programs across multiple state systems. Adm Policy Ment Hlth.

[26] Substance Abuse and Mental Health Services Administration (2011). Substance abuse and mental health services administration, dual diagnosis capability in addiction treatment toolkit version 4.0.

[27] Gertner AK, Clare HM, Powell BJ, Gilbert AR, Jones HE, Silberman P (2022). A mixed methods study of provider factors in buprenorphine treatment retention. Int J Drug Policy..

[28] McNary AL (2021). Myths and misconceptions: terminating treatment. Innov Clin Neurosci.

[29] Thomas KC, Ellis AR, Konrad TR, Holzer CE, Morrissey JP (2009). County-level estimates of mental health professional shortage in the United States. Psychiat Serv.

[30] McLean CP, Asnaani A, Litz BT, Hofmann SG (2011). Gender differences in anxiety disorders: prevalence, course of illness, comorbidity and burden of illness. J Psychiatr Res.

[31] Barbosa-Leiker C, Campbell ANC, McHugh RK, Guille C, Greenfield SF (2021). Opioid use disorder in women and the implications for treatment. Psychiatr Res Clin Pract.

[32] Stout MJ, Bedrick B, O'Donnell C, Hernandez J, Carter EB, Kelly J (2020). Access to buprenorphine in pregnancy: minimizing barriers for patients. Am J Obstet Gynecol.

[33] Xu KY, Jones HE, Schiff DM, Martin CE, Kelly JC, Carter EB (2023). Initiation and treatment discontinuation of medications for opioid use disorder in pregnant people compared with nonpregnant people. Obstet Gynecol.

[34] Xu KY, Schiff DM, Jones HE, Martin CE, Kelly JC, Bierut LJ (2023). Racial and ethnic inequities in buprenorphine and methadone utilization among reproductive-age women with opioid use disorder: an analysis of multi-state medicaid claims in the USA. J Gen Intern Med..

[35] Schiff DM, Nielsen TC, Hoeppner BB, Terplan M, Hadland SE, Bernson D (2021). Methadone and buprenorphine discontinuation among postpartum women with opioid use disorder. Am J Obstet Gynecol..

[36] Greenfield S, Rosa C, Barbosa-Leiker C, Campbell A, McHugh K, Guille C (2019). Opioid Use Disorder in Women: Evidence from the National Institute on Drug Abuse Clinical Trials Network (CTN) and the Implications for Treatment. Am J Addictn..

[37] Fornili KS, Fogger SA (2017). Nurse practitioner prescriptive authority for buprenorphine: from DATA 2000 to CARA 2016. J Addict Nurs.

