# Supplementary material for: Treatment setting and buprenorphine discontinuation: an analysis of multi-state insurance claims
Source: Addict Sci Clin Pract. 2024 Mar 16;19:17. doi: 10.1186/s13722-024-00450-0 (PMC10943881; doi:10.1186/s13722-024-00450-0)
Supplement: Supplementary file 1 — Additional file 1: Methods S1. Design Diagram. Table S1. Classification of Setting Type. Table S2. Administrative codes for classification of covariates. Table S3. Treatment Setting Type and Time to Buprenorphine Discontinuation, Sensitivity Analyses by Length of Buprenorphine Episode Gap. Table S4. Treatment Setting Type and Time to Buprenorphine Discontinuation, Sensitivity Analyses by Length of Prescription-Prescriber Visit Linkage. Table S5. Treatment Setting Type and Time to Buprenorphine Discontinuation, Controlling for Mean Buprenorphine Dose During Treatment Episode. Table S6. Insurance status, Setting, and 180-day and 365-day buprenorphine retention. [file 13722_2024_450_MOESM1_ESM.docx]

**Additional file 1**

Methods: Design Diagram

Table S1: Classification of Setting Type

Table S2: Administrative codes for classification of covariates

Table S3: Treatment Setting Type and Time to Buprenorphine Discontinuation, Sensitivity Analyses by Length of Buprenorphine Episode Gap

Table S4: Treatment Setting Type and Time to Buprenorphine Discontinuation, Sensitivity Analyses by Length of Prescription-Prescriber Visit Linkage

Table S5: Treatment Setting Type and Time to Buprenorphine Discontinuation, Controlling for Mean Buprenorphine Dose During Treatment Episode

Table S6: Insurance status, Setting, and 180-day and 365-day buprenorphine retention

Methods: Design Diagram

Patients were followed until **the discontinuation of buprenorphine**

INDEX EVENT

TIME=0

First episode of

buprenorphine

**“Bup_start”**

Start of insurance enrollment or January 1, 2006

**“Elig_start”**

End of insurance enrollment or December 31, 2016

**“Elig_end”**

For descriptive purposes, we computed the % of individuals who were retained in treatment at (Bup_start PLUS 6 months) and (Bup_start PLUS 1 year )

Bup_start

MINUS 6 months

Bup_start

PLUS 30 days

Bup_start

MINUS 14 days

|----------------|

Linkage of buprenorphine scripts with provider visits in 14 days prior to Bup_start (with sensitivity analysis done for 30 days prior to Bup_start)

|-----------------------------------------|

Ascertainment of covariates

Between >6 months

pre-buprenorphine initiation

Table S1: Classification of Setting Type

| Claim type | Definition |
| --- | --- |
| Inpatient | SVCSCAT not ending with “20” |
|  | AND |
|  | STDPLAC=21 or ( STDPLAC not 23 and SVCSCAT=10xxx, 20xxx, 301-303xx, 311-313xx, 22130-22132, 22135-22137, 22140, 22115, 22120, 22126, 22141, 22151-22156, 22159, 22161-22169, or 22199) |
| Emergency department | Not an inpatient claim (defined above) |
|  | AND |
|  | STDPLAC=23 or SVCSCAT ending with “20” |
| Prescriber visit in outpatient settings (i.e., Office visit) | Not an inpatient or emergency department claim (defined above) |
|  | AND |
|  | STDPLAC=11, 18-19, 22, 49-50, 71-72, 95, or 99 |
|  | AND |
|  | (SVCSCAT=xxx24-xxx26, 21199, 21299, 22399, 12328, or 12399) or (SVCSCAT=30518 AND STDPROV=365, 240, or 200 AND Proc1=90862) or (SVCSCAT=31518 AND STDPROV=365, 240, or 200 AND Proc1=90807 or 90862-90863) or (SVCSCAT=22325 AND STDPROV=825, 845, or 822 AND Proc1=99213-99214) |
|  |  |

Classification of setting type using 14 days as a threshold for linking buprenorphine fills with provider visits (**primary analysis)**

| Outpatient Primary Care n=23,899 people receiving buprenorphine from outpatient primary care | Outpatient visit (defined above) |
| --- | --- |
|  | AND |
|  | STDPROV=40, 202, 204, 400, or 245 |
|  |  |
|  | **22,261 were in the outpatient primary care cohort, as outpatient primary care constituted the most common setting type (based on number of encounters) during the 14 days before treatment initiation** |
|  | **1,638 who had a tie among the most common treatment setting (based on number of encounters) and were classified in the outpatient primary care medicine cohort because they initiated treatment in outpatient primary care medicine** |
|  | 207 for whom the most common treatment setting was a single tie for outpatient primary care and outpatient psychiatry |
|  | 1,388 for whom the most common treatment setting was a single tie for outpatient primary care and SUD facility |
|  | 43 who had initiated treatment with outpatient primary care and had multiple ties across primary care and SUD facility treatment settings |
|  |  |
| Outpatient Psychiatry , n=8,133 people receiving buprenorphine from outpatient psychiatrists | Outpatient visit (defined above) |
|  | AND |
|  | STDPROV=365, 458 |
|  |  |
|  | **7,074 were in the outpatient psychiatry cohort, as outpatient psychiatry constituted the most common setting type (based on number of encounters) during the 14 days before treatment initiation** |
|  | **1,059 who had a tie among the most common treatment setting (based on number of encounters) and were classified in the outpatient psychiatry cohort because they initiated treatment in outpatient psychiatry** |
|  | 490 for whom the most common treatment setting was a single tie for outpatient primary care and outpatient psychiatry |
|  | 501 for whom the most common treatment setting was a single tie for outpatient psychiatry and SUD facility |
|  | 68 who had initiated treatment with outpatient psychiatry and had multiple ties across primary care and SUD facilities |
|  |  |
| SUD facility n= 26,168 people receiving buprenorphine from SUD treatment facilities | Not an inpatient ,emergency department, or outpatient visit (defined above) |
|  | AND |
|  | (SVCSCAT=30524-30526, 30624-30626, 31524-31526, 31624-31626) or STDPLAC=55 or 56 or (proc1=H0017-H0019) or (SVCSCAT=22325 AND STDPROV=40 AND Proc1=99213-99214) or (SVCSCAT=30618 AND STDPROV=21-22 or 40 AND Proc1=90807 or 90862-90863) |
|  | **23,089 were in the primary care cohort, as SUD treatment facilities constituted the most common setting type (based on number of encounters) during the 14 days before treatment initiation** |
|  | **3,079 who had a tie among the most common treatment setting (based on number of encounters) and were classified in the SUD facility cohort because they initiated treatment in an SUD facility** |
|  | 2,369 for whom the most common treatment setting was a single tie for outpatient primary care and SUD facility |
|  | 631 for whom the most common treatment setting was a single tie for outpatient psychiatry and SUD facility |
|  | 79 who had initiated treatment with SUD facilities and had multiple ties across primary care and outpatient facilities |
|  |  |

Classification of setting type using 30 days as a threshold for linking buprenorphine fills with provider visits (**secondary analysis in Table S4)**

| Claim type | Definition |
| --- | --- |
| Outpatient Primary Care n=24,079 people receiving buprenorphine from outpatient primary care | Outpatient visit (defined above) |
|  | AND |
|  | STDPROV=40, 202, 204, 400, or 245 |
|  |  |
|  | **22,579 were in the outpatient primary care cohort, as outpatient primary care constituted the most common setting type (based on number of encounters) during the 30 days before treatment initiation** |
|  | **1,500 who had a tie among the most common treatment setting (based on number of encounters) and were classified in the outpatient primary care medicine cohort because they initiated treatment in outpatient primary care medicine** |
|  | 184 for whom the most common treatment setting was a single tie for outpatient primary care and outpatient psychiatry |
|  | 1,271 for whom the most common treatment setting was a single tie for outpatient primary care and SUD facility |
|  | 45 who had initiated treatment with outpatient primary care and had multiple ties across primary care and SUD facility treatment settings |
|  |  |
| Outpatient Psychiatry , n=8,142 people receiving buprenorphine from outpatient psychiatrists | Outpatient visit (defined above) |
|  | AND |
|  | STDPROV=365, 458 |
|  |  |
|  | **7,170 were in the outpatient psychiatry cohort, as outpatient psychiatry constituted the most common setting type (based on number of encounters) during the 30 days before treatment initiation** |
|  | **972 who had a tie among the most common treatment setting (based on number of encounters) and were classified in the outpatient psychiatry cohort because they initiated treatment in outpatient psychiatry** |
|  | 445 for whom the most common treatment setting was a single tie for outpatient primary care and outpatient psychiatry |
|  | 473 for whom the most common treatment setting was a single tie for outpatient psychiatry and SUD facility |
|  | 41 who had initiated treatment with outpatient psychiatry and had multiple ties across primary care and SUD facilities |
|  |  |
| SUD facility, n= 25,979 people receiving buprenorphine from SUD treatment facilities | Not an inpatient ,emergency department, or outpatient visit (defined above) |
|  | AND |
|  | (SVCSCAT=30524-30526, 30624-30626, 31524-31526, 31624-31626) or STDPLAC=55 or 56 or (proc1=H0017-H0019) or (SVCSCAT=22325 AND STDPROV=40 AND Proc1=99213-99214) or (SVCSCAT=30618 AND STDPROV=21-22 or 40 AND Proc1=90807 or 90862-90863) |
|  | **23,203 were in the primary care cohort, as SUD treatment facilities constituted the most common setting type (based on number of encounters) during the 30 days before treatment initiation** |
|  | **2,776 who had a tie among the most common treatment setting (based on number of encounters) and were classified in the SUD facility cohort because they initiated treatment in an SUD facility** |
|  | 2,129 for whom the most common treatment setting was a single tie for outpatient primary care and SUD facility |
|  | 582 for whom the most common treatment setting was a single tie for outpatient psychiatry and SUD facility |
|  | 46 who had initiated treatment with SUD facilities and had multiple ties across primary care and outpatient facilities |
|  |  |

Table S2: Administrative codes for classification of covariates

|  | Administrative Codes, ascertained in the 6 months preceding buprenorphine initiation through buprenorphine initiation (time=0, Index date) |
| --- | --- |
| Admissions for drug-related poisonings | Any one of the following ICD-9/10 codes for drug-related poisonings: T40, T41, T42, T43, T44, T45, T46, T47, T48, T49, T50, T51, T52, T53, T54, T55, T56, T57, T58, T59, T60, T61, T62, T63, T64, T65, 960, 961, 962, 963, 964, 965, 966, 967, 968, 969, 970, 971, 972, 973, 974, 975, 976, 977, 978, 979, 980, 981, 982, 983, 984, 985, 986, 987, 988, 989, E850, E851, E852, E853, E854, E855, E856, E857, E858, E860, E861, E862, E863, E864, E865, E866, E867, E868, E869, E950, E951, E952, E962, E972, E975, E976, E980, E981, E982 |
| Alcohol Use Disorder | Any one of the following ICD-9/10 codes: 303.9, 305.0, F10 |
| Sedative Use Disorder | Any one of the following ICD-9/10 codes: 304.1, 305.4: F13 |
| Cocaine Use Disorder | Any one of the following ICD-9/10 codes: 305.6, 304.2, F14 |
| Methamphetamine Use Disorder | Any one of the following ICD-9/10 codes: 305.7, 304.4, F15 |
| Anxiety Disorders | Any one of the following ICD-9/10 codes: 300.0-316.9, F40-F49 |
| Mood Disorder | Any one of the following ICD-9/10 codes: 296.0-296.9, F30-F39 |
| Psychotic Disorders | Any one of the following ICD-9/10 codes: 295.0-295.9, F20-F29 |

Table S3: Treatment Setting Type and Time to Buprenorphine Discontinuation, Sensitivity Analyses by Length of Buprenorphine Episode Gap

|  | 45 day gaps (Model 1 in Table 2) | | | 30 day gaps | | | 60 day gaps | | |
| --- | --- | --- | --- | --- | --- | --- | --- | --- | --- |
|  | aHR | 95% CI | | aHR | 95% CI | | aHR | 95% CI | |
| Substance Use Disorder Facility vs. Outpatient Psychiatry | 1.03 | 1.01 | 1.06 | 1.02 | 0.99 | 1.05 | 1.04 | 1.01 | 1.07 |
| Outpatient Primary Care vs Outpatient Psychiatry | 1.08 | 1.05 | 1.10 | 1.08 | 1.05 | 1.11 | 1.06 | 1.03 | 1.09 |
| Male vs Female | 1.02 | 1.00 | 1.04 | 1.01 | 0.99 | 1.03 | 1.02 | 1.01 | 1.04 |
| Commercial vs Medicaid | 0.80 | 0.78 | 0.82 | 0.83 | 0.81 | 0.85 | 0.81 | 0.79 | 0.83 |
| Age > 30 vs < 30 years | 0.76 | 0.75 | 0.77 | 0.77 | 0.76 | 0.79 | 0.75 | 0.74 | 0.77 |
| Co-occurring AUD vs no AUD | 1.05 | 1.02 | 1.09 | 1.05 | 1.02 | 1.09 | 1.07 | 1.03 | 1.10 |
| Co-occurring MUD vs no MUD | 1.17 | 1.11 | 1.24 | 1.17 | 1.11 | 1.24 | 1.19 | 1.13 | 1.26 |
| Co-occurring CUD vs no CUD | 1.21 | 1.16 | 1.26 | 1.21 | 1.16 | 1.27 | 1.21 | 1.16 | 1.26 |
| Co-occurring XUD vs no XUD | 1.09 | 1.05 | 1.14 | 1.08 | 1.04 | 1.12 | 1.10 | 1.06 | 1.14 |
| Co-occurring mood d/o vs no mood d/o | 1.08 | 1.06 | 1.10 | 1.09 | 1.06 | 1.11 | 1.08 | 1.06 | 1.10 |
| Co-occurring anxiety d/o vs no anxiety d/o | 1.05 | 1.03 | 1.07 | 1.05 | 1.03 | 1.07 | 1.05 | 1.03 | 1.07 |
| Co-occurring psychotic d/o vs no psychotic d/o | 1.05 | 0.98 | 1.12 | 1.03 | 0.96 | 1.10 | 1.06 | 0.99 | 1.13 |
| CCI=1 or 2 vs CCI=0 | 1.06 | 1.03 | 1.09 | 1.05 | 1.02 | 1.09 | 1.07 | 1.04 | 1.10 |
| CCI=3+ vs CCI=0 | 1.05 | 0.95 | 1.15 | 1.06 | 0.96 | 1.16 | 1.07 | 0.97 | 1.17 |
| Drug-related poisoning in the 6 months preceding buprenorphine initiation (Yes v No) | 1.13 | 1.10 | 1.17 | 1.13 | 1.10 | 1.17 | 1.16 | 1.12 | 1.19 |

The models below control for race/ethnicity, which is ONLY available among Medicaid enrollees, and thus the following models were only conducted in the Medicaid subset of the sample.

|  | 45 day gaps (Model 2 in Table 2) | | | 30 day gaps | | | 60 day gaps | | |
| --- | --- | --- | --- | --- | --- | --- | --- | --- | --- |
|  | aHR | 95% CI | | aHR | 95% CI | | aHR | 95% CI | |
| Substance Use Disorder Facility vs. Outpatient Psychiatry | 1.21 | 1.12 | 1.30 | 1.18 | 1.09 | 1.27 | 1.21 | 1.12 | 1.31 |
| Outpatient Primary Care vs Outpatient Psychiatry | 1.36 | 1.26 | 1.47 | 1.33 | 1.23 | 1.44 | 1.29 | 1.19 | 1.39 |
| Male vs Female | 1.15 | 1.10 | 1.20 | 1.14 | 1.09 | 1.19 | 1.16 | 1.11 | 1.21 |
| Non Hispanic Black vs Non Hispanic White | 1.03 | 0.95 | 1.12 | 1.01 | 0.93 | 1.10 | 1.05 | 0.97 | 1.14 |
| Hispanic vs Non Hispanic White | 1.00 | 0.85 | 1.18 | 1.02 | 0.87 | 1.20 | 1.02 | 0.86 | 1.19 |
| Other Race vs Non Hispanic White | 0.84 | 0.79 | 0.88 | 0.86 | 0.81 | 0.91 | 0.87 | 0.82 | 0.92 |
| Age > 30 vs < 30 years | 0.86 | 0.83 | 0.90 | 0.87 | 0.83 | 0.90 | 0.86 | 0.83 | 0.90 |
| Co-occurring AUD vs no AUD | 1.05 | 0.98 | 1.12 | 1.06 | 0.99 | 1.14 | 1.04 | 0.97 | 1.12 |
| Co-occurring MUD vs no MUD | 1.09 | 1.00 | 1.20 | 1.11 | 1.02 | 1.22 | 1.14 | 1.04 | 1.25 |
| Co-occurring CUD vs no CUD | 1.27 | 1.18 | 1.36 | 1.30 | 1.21 | 1.40 | 1.28 | 1.19 | 1.38 |
| Co-occurring XUD vs no XUD | 1.12 | 1.03 | 1.22 | 1.10 | 1.01 | 1.20 | 1.13 | 1.04 | 1.23 |
| Co-occurring mood d/o vs no mood d/o | 1.03 | 0.99 | 1.07 | 1.03 | 0.99 | 1.08 | 1.03 | 0.99 | 1.07 |
| Co-occurring anxiety d/o vs no anxiety d/o | 1.02 | 0.98 | 1.06 | 1.02 | 0.98 | 1.06 | 1.01 | 0.97 | 1.05 |
| Co-occurring psychotic d/o vs no psychotic d/o | 0.98 | 0.88 | 1.09 | 0.97 | 0.86 | 1.08 | 0.98 | 0.87 | 1.09 |
| CCI=1 or 2 vs CCI=0 | 1.00 | 0.94 | 1.06 | 0.98 | 0.92 | 1.04 | 1.03 | 0.97 | 1.10 |
| CCI=3+ vs CCI=0 | 0.92 | 0.79 | 1.08 | 0.92 | 0.78 | 1.07 | 0.95 | 0.81 | 1.11 |
| Drug-related poisoning in the 6 months preceding buprenorphine initiation (Yes v No) | 1.22 | 1.14 | 1.30 | 1.21 | 1.14 | 1.29 | 1.26 | 1.19 | 1.35 |

AUD=alcohol use disorder, CCI=Charlson comorbidity index, d/o=disorder, CUD=cocaine use disorder, MUD=amphetamine use disorder, =substance use disorder, XUD=sedative use disorder

Table S4: Treatment Setting Type and Time to Buprenorphine Discontinuation, Sensitivity Analyses by Length of Prescription-Prescriber Visit Linkage

The detailed linkage scheme for 14-day linkage and 30-day linkage can be seen in eTable 1.

|  | 14 day linkage (Model 1 in Table 2) | | | 30 day linkage | | |
| --- | --- | --- | --- | --- | --- | --- |
|  | aHR | 95% CI | | aHR | 95% CI | |
| Substance Use Disorder Facility vs. Outpatient Psychiatry | 1.03 | 1.01 | 1.06 | 1.03 | 1.01 | 1.06 |
| Outpatient Primary Care vs Outpatient Psychiatry | 1.08 | 1.05 | 1.10 | 1.08 | 1.05 | 1.10 |
| Male vs Female | 1.02 | 1.00 | 1.04 | 1.02 | 1.00 | 1.04 |
| Commercial vs Medicaid | 0.80 | 0.78 | 0.82 | 0.80 | 0.78 | 0.82 |
| Age > 30 vs < 30 years | 0.76 | 0.75 | 0.77 | 0.76 | 0.75 | 0.77 |
| Co-occurring AUD vs no AUD | 1.05 | 1.02 | 1.09 | 1.05 | 1.02 | 1.08 |
| Co-occurring MUD vs no MUD | 1.17 | 1.11 | 1.24 | 1.17 | 1.11 | 1.24 |
| Co-occurring CUD vs no CUD | 1.21 | 1.16 | 1.26 | 1.21 | 1.16 | 1.26 |
| Co-occurring XUD vs no XUD | 1.09 | 1.05 | 1.14 | 1.09 | 1.05 | 1.14 |
| Co-occurring mood d/o vs no mood d/o | 1.08 | 1.06 | 1.10 | 1.08 | 1.06 | 1.10 |
| Co-occurring anxiety d/o vs no anxiety d/o | 1.05 | 1.03 | 1.07 | 1.05 | 1.03 | 1.07 |
| Co-occurring psychotic d/o vs no psychotic d/o | 1.05 | 0.98 | 1.12 | 1.04 | 0.98 | 1.12 |
| CCI=1 or 2 vs CCI=0 | 1.06 | 1.03 | 1.09 | 1.06 | 1.03 | 1.09 |
| CCI=3+ vs CCI=0 | 1.05 | 0.95 | 1.15 | 1.05 | 0.95 | 1.15 |
| Drug-related poisoning in the 6 months preceding buprenorphine initiation (Yes v No) | 1.13 | 1.10 | 1.17 | 1.14 | 1.11 | 1.17 |

The models below control for race/ethnicity, which is ONLY available among Medicaid enrollees, and thus the following models were only conducted in the Medicaid subset of the sample.

|  | 14 day linkage (Model 2 in Table 2) | | | 30 day linkage | | |
| --- | --- | --- | --- | --- | --- | --- |
|  | aHR | 95% CI | | aHR | 95% CI | |
| Substance Use Disorder Facility vs. Outpatient Psychiatry | 1.21 | 1.12 | 1.30 | 1.19 | 1.10 | 1.28 |
| Outpatient Primary Care vs Outpatient Psychiatry | 1.36 | 1.26 | 1.47 | 1.34 | 1.24 | 1.44 |
| Male vs Female | 1.15 | 1.10 | 1.20 | 1.15 | 1.10 | 1.20 |
| Non Hispanic Black vs Non Hispanic White | 1.03 | 0.95 | 1.12 | 1.03 | 0.95 | 1.12 |
| Hispanic vs Non Hispanic White | 1.00 | 0.85 | 1.18 | 1.00 | 0.85 | 1.18 |
| Other Race vs Non Hispanic White | 0.84 | 0.79 | 0.88 | 0.84 | 0.79 | 0.89 |
| Age > 30 vs < 30 years | 0.86 | 0.83 | 0.90 | 0.86 | 0.83 | 0.90 |
| Co-occurring AUD vs no AUD | 1.05 | 0.98 | 1.12 | 1.05 | 0.98 | 1.12 |
| Co-occurring MUD vs no MUD | 1.09 | 1.00 | 1.20 | 1.09 | 1.00 | 1.20 |
| Co-occurring CUD vs no CUD | 1.27 | 1.18 | 1.36 | 1.27 | 1.18 | 1.36 |
| Co-occurring XUD vs no XUD | 1.12 | 1.03 | 1.22 | 1.12 | 1.03 | 1.22 |
| Co-occurring mood d/o vs no mood d/o | 1.03 | 0.99 | 1.07 | 1.02 | 0.98 | 1.06 |
| Co-occurring anxiety d/o vs no anxiety d/o | 1.02 | 0.98 | 1.06 | 1.02 | 0.98 | 1.07 |
| Co-occurring psychotic d/o vs no psychotic d/o | 0.98 | 0.88 | 1.09 | 0.98 | 0.87 | 1.09 |
| CCI=1 or 2 vs CCI=0 | 1.00 | 0.94 | 1.06 | 1.00 | 0.94 | 1.06 |
| CCI=3+ vs CCI=0 | 0.92 | 0.79 | 1.08 | 0.93 | 0.79 | 1.08 |
| Drug-related poisoning in the 6 months preceding buprenorphine initiation (Yes v No) | 1.22 | 1.14 | 1.30 | 1.23 | 1.15 | 1.30 |

AUD=alcohol use disorder, CCI=Charlson comorbidity index, d/o=disorder, CUD=cocaine use disorder, MUD=amphetamine use disorder, =substance use disorder, XUD=sedative use disorder

Table S5: Treatment Setting Type and Time to Buprenorphine Discontinuation, Controlling for Mean Buprenorphine Dose During Treatment Episode

|  | Model 1 in Table 2 | | |  |  | | |
| --- | --- | --- | --- | --- | --- | --- | --- |
|  | aHR | 95% CI | |  | aHR | 95% CI | |
| Substance Use Disorder Facility vs. Outpatient Psychiatry | 1.03 | 1.01 | 1.06 | Substance Use Disorder Facility vs. Outpatient Psychiatry | 1.03 | 1.01 | 1.06 |
| Outpatient Primary Care vs Outpatient Psychiatry | 1.08 | 1.05 | 1.10 | Outpatient Primary Care vs Outpatient Psychiatry | 1.08 | 1.05 | 1.11 |
| Male vs Female | 1.02 | 1.00 | 1.04 | Male vs Female | 1.02 | 1.00 | 1.04 |
| Commercial vs Medicaid | 0.80 | 0.78 | 0.82 | Commercial vs Medicaid | 0.80 | 0.78 | 0.82 |
| Age > 30 vs < 30 years | 0.76 | 0.75 | 0.77 | Age > 30 vs < 30 years | 0.76 | 0.75 | 0.77 |
| Co-occurring AUD vs no AUD | 1.05 | 1.02 | 1.09 | Co-occurring AUD vs no AUD | 1.05 | 1.02 | 1.08 |
| Co-occurring MUD vs no MUD | 1.17 | 1.11 | 1.24 | Co-occurring MUD vs no MUD | 1.17 | 1.11 | 1.24 |
| Co-occurring CUD vs no CUD | 1.21 | 1.16 | 1.26 | Co-occurring CUD vs no CUD | 1.21 | 1.16 | 1.26 |
| Co-occurring XUD vs no XUD | 1.09 | 1.05 | 1.14 | Co-occurring XUD vs no XUD | 1.09 | 1.05 | 1.14 |
| Co-occurring mood d/o vs no mood d/o | 1.08 | 1.06 | 1.10 | Co-occurring mood d/o vs no mood d/o | 1.08 | 1.06 | 1.10 |
| Co-occurring anxiety d/o vs no anxiety d/o | 1.05 | 1.03 | 1.07 | Co-occurring anxiety d/o vs no anxiety d/o | 1.05 | 1.03 | 1.07 |
| Co-occurring psychotic d/o vs no psychotic d/o | 1.05 | 0.98 | 1.12 | Co-occurring psychotic d/o vs no psychotic d/o | 1.05 | 0.98 | 1.12 |
| CCI=1 or 2 vs CCI=0 | 1.06 | 1.03 | 1.09 | CCI=1 or 2 vs CCI=0 | 1.06 | 1.03 | 1.09 |
| CCI=3+ vs CCI=0 | 1.05 | 0.95 | 1.15 | CCI=3+ vs CCI=0 | 1.05 | 0.96 | 1.15 |
| Drug-related poisoning in the 6 months preceding buprenorphine initiation | 1.13 | 1.10 | 1.17 | Mean daily dose of 16+ mg of Buprenorphine (Yes v No) | 0.95 | 0.93 | 0.97 |
|  |  |  |  | Mean daily dose of 24+ mg of Buprenorphine (Yes v No) | 1.00 | 0.96 | 1.04 |
|  |  |  |  | Drug-related poisoning in the 6 months preceding buprenorphine initiation (Yes v No) | 1.13 | 1.10 | 1.16 |

The models below control for race/ethnicity, which is ONLY available among Medicaid enrollees, and thus the following models were only conducted in the Medicaid subset of the sample.

|  | Model 2 in Table 2 | | |  |  | | |
| --- | --- | --- | --- | --- | --- | --- | --- |
|  | aHR | 95% CI | |  | aHR | 95% CI | |
| Substance Use Disorder Facility vs. Outpatient Psychiatry | 1.21 | 1.12 | 1.30 | Substance Use Disorder Facility vs. Outpatient Psychiatry | 1.19 | 1.10 | 1.28 |
| Outpatient Primary Care vs Outpatient Psychiatry | 1.36 | 1.26 | 1.47 | Outpatient Primary Care vs Outpatient Psychiatry | 1.34 | 1.24 | 1.44 |
| Male vs Female | 1.15 | 1.10 | 1.20 | Male vs Female | 1.15 | 1.11 | 1.20 |
| Non Hispanic Black vs Non Hispanic White | 1.03 | 0.95 | 1.12 | Non Hispanic Black vs Non Hispanic White | 1.02 | 0.94 | 1.10 |
| Hispanic vs Non Hispanic White | 1.00 | 0.85 | 1.18 | Hispanic vs Non Hispanic White | 0.98 | 0.83 | 1.15 |
| Other Race vs Non Hispanic White | 0.84 | 0.79 | 0.88 | Other Race vs Non Hispanic White | 0.83 | 0.78 | 0.88 |
| Age > 30 vs < 30 years | 0.86 | 0.83 | 0.90 | Age > 30 vs < 30 years | 0.87 | 0.84 | 0.91 |
| Co-occurring AUD vs no AUD | 1.05 | 0.98 | 1.12 | Co-occurring AUD vs no AUD | 1.05 | 0.98 | 1.13 |
| Co-occurring MUD vs no MUD | 1.09 | 1.00 | 1.20 | Co-occurring MUD vs no MUD | 1.09 | 0.99 | 1.19 |
| Co-occurring CUD vs no CUD | 1.27 | 1.18 | 1.36 | Co-occurring CUD vs no CUD | 1.26 | 1.17 | 1.36 |
| Co-occurring XUD vs no XUD | 1.12 | 1.03 | 1.22 | Co-occurring XUD vs no XUD | 1.12 | 1.03 | 1.22 |
| Co-occurring mood d/o vs no mood d/o | 1.03 | 0.99 | 1.07 | Co-occurring mood d/o vs no mood d/o | 1.02 | 0.98 | 1.06 |
| Co-occurring anxiety d/o vs no anxiety d/o | 1.02 | 0.98 | 1.06 | Co-occurring anxiety d/o vs no anxiety d/o | 1.02 | 0.98 | 1.07 |
| Co-occurring psychotic d/o vs no psychotic d/o | 0.98 | 0.88 | 1.09 | Co-occurring psychotic d/o vs no psychotic d/o | 0.98 | 0.88 | 1.10 |
| CCI=1 or 2 vs CCI=0 | 1.00 | 0.94 | 1.06 | CCI=1 or 2 vs CCI=0 | 1.01 | 0.95 | 1.07 |
| CCI=3+ vs CCI=0 | 0.92 | 0.79 | 1.08 | CCI=3+ vs CCI=0 | 0.92 | 0.79 | 1.08 |
| Drug-related poisoning in the 6 months preceding buprenorphine initiation (Yes v No) | 1.22 | 1.14 | 1.30 | Mean daily dose of 16+ mg of Buprenorphine (Yes v No) | 0.80 | 0.76 | 0.85 |
|  |  |  |  | Mean daily dose of 24+ mg of Buprenorphine (Yes v No) | 1.24 | 1.13 | 1.36 |
|  |  |  |  | Drug-related poisoning in the 6 months preceding buprenorphine initiation (Yes v No) | 1.21 | 1.14 | 1.29 |

AUD=alcohol use disorder, CCI=Charlson comorbidity index, d/o=disorder, CUD=cocaine use disorder, MUD=amphetamine use disorder, =substance use disorder, XUD=sedative use disorder

Table S6: Insurance status, Setting, and 180-day and 365-day buprenorphine retention

|  | 180-day retention | | p | 365-day retention | | p |
| --- | --- | --- | --- | --- | --- | --- |
|  | No | Yes |  | No | Yes |  |
|  |  |  | <.001 |  |  | <.001 |
| Commercial Insurance, n=45,913 | 22,711 (49.5) | 23,202 (50.5) |  | 32,772 (71.4) | 13,141 (28.6) |  |
| Medicaid , n=12,287 | 6,712 (54.6) | 5,575 (45.4) |  | 9,881 (80.4) | 2,406 (19.6) |  |

|  | 180-day retention | | p | 365-day retention | | p |
| --- | --- | --- | --- | --- | --- | --- |
|  | No | Yes |  | No | Yes |  |
|  |  |  | <.001 |  |  | <.001 |
| SUD Facility + Medicaid , n=6,640 | 3,521 (53.0) | 3119 (47.0) |  | 5,302 (79.9) | 1,338 (20.2) |  |
| SUD Facility + Commercial Insurance , n=19,528 | 9,608 (49.2) | 9,920 (50.8) |  | 14,021 (71.8) | 5,507 (28.2) |  |
| Outpatient Primary Care + Medicaid, n=4,841 | 2,798 (57.8) | 2,043 (42.2) |  | 4,010 (82.8) | 831 (17.2) |  |
| Outpatient Primary Care + Commercial Insurance , n=19,058 | 9,410 (49.4) | 9,648 (50.6) |  | 13,535 (71.0) | 5,523 (29.0) |  |
| Outpatient Psychiatry + Medicaid , n=806 | 393 (48.8) | 413 (51.2) |  | 569 (70.6) | 237 (29.4) |  |
| Outpatient Psychiatry + Commercial Insurance, n=7,327 | 3,693 (50.4) | 3,634 (49.6) |  | 5,216 (71.2) | 2,111 (28.8) |  |
